# Supplementary material for: Microbial Transformation of Licochalcones
Source: Molecules. 2019 Dec 23;25(1):60. doi: 10.3390/molecules25010060 (PMC6982849; doi:10.3390/molecules25010060)
Supplement: Supplementary file 1 [file molecules-25-00060-s001.pdf]

Supplementary Materials

Microbial transformation of licochalcones

Yina Xiao, Fubo Han and Ik-Soo Lee *

College of Pharmacy, Chonnam National University, Gwangju 61186, Korea

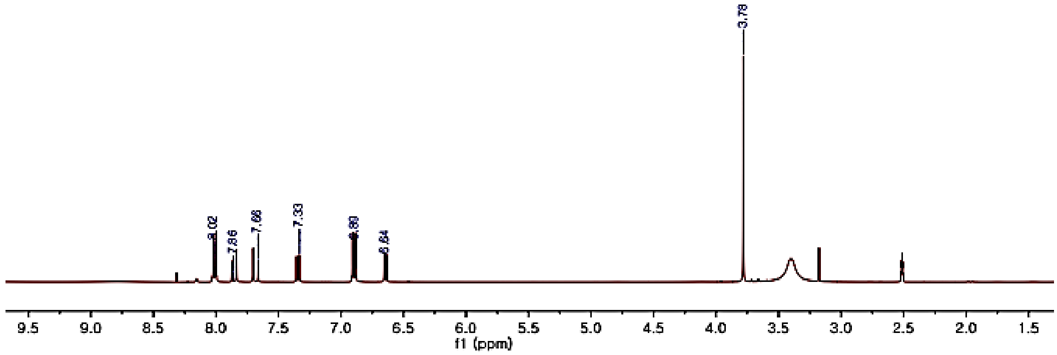

Figure S1. ^{1}H NMR spectrum of licochalcone B (**1**) (DMSO-d_6)

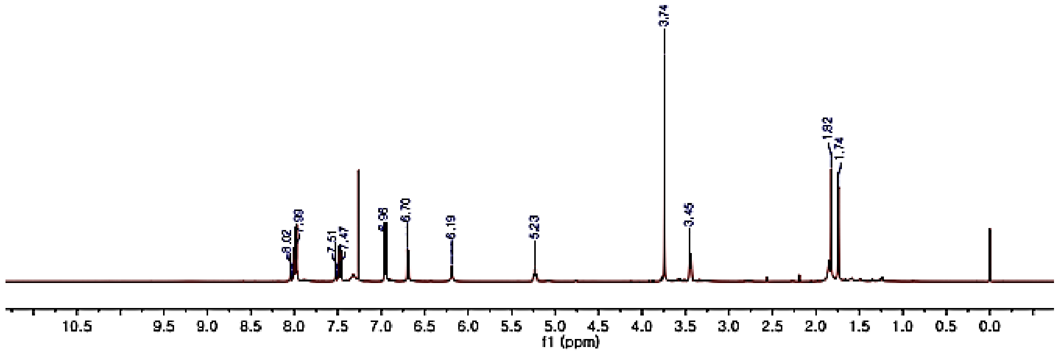

Figure S2. ^{1}H NMR spectrum of licochalcone C (**2**) ($CDCl_3$)

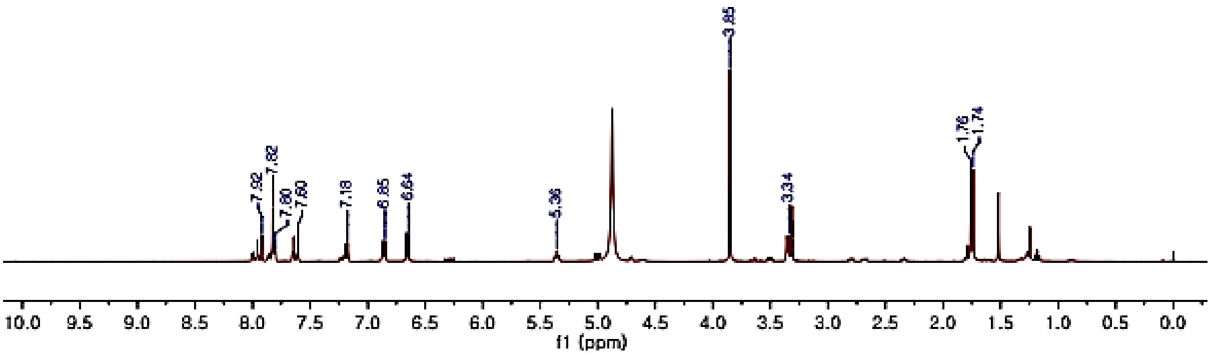

Figure S3. ^{1}H NMR spectrum of licochalcone D (**3**) (CD_3OD)

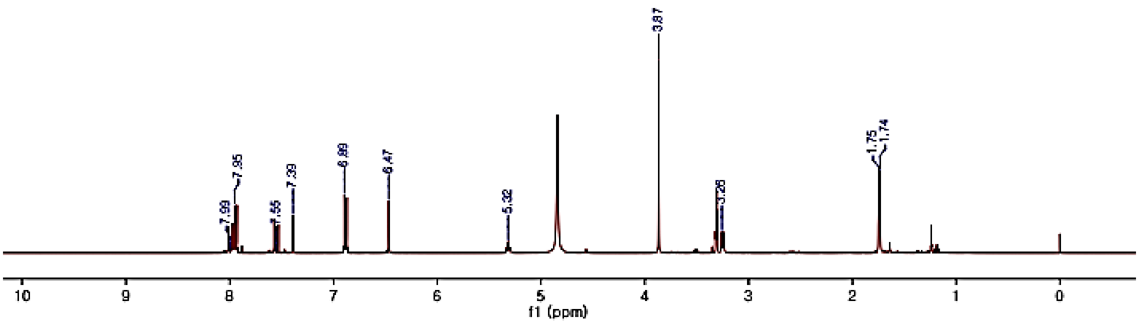

Figure S4. ^{1}H NMR spectrum of licochalcone H (**4**) (CD_3OD)

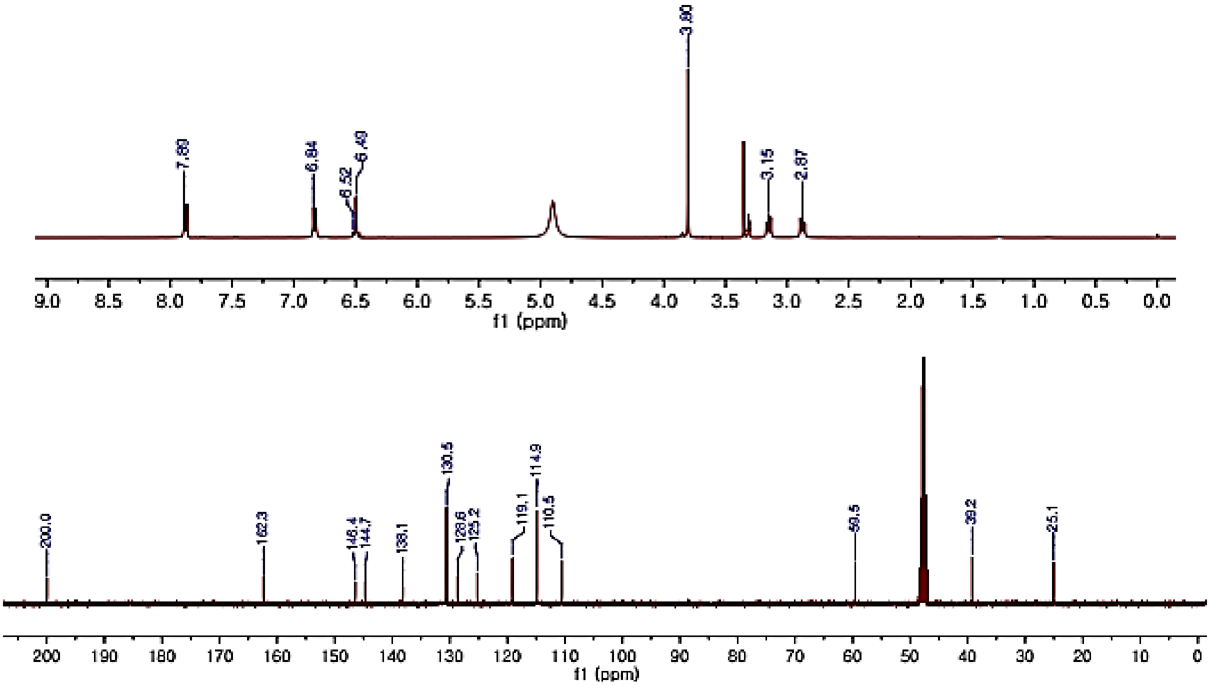

Figure S5. ^{1}H and ^{13}C NMR spectra of metabolite **5** (CD_3OD)

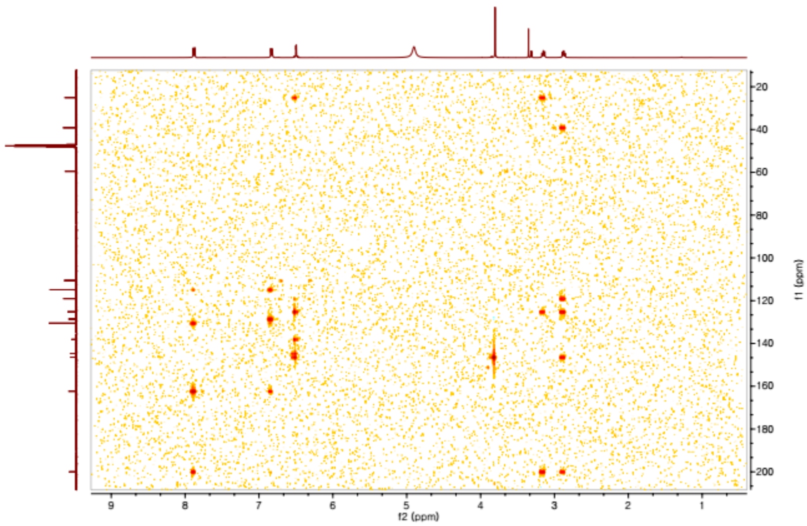

Figure S6. HMBC of metabolite **5** (CD_3OD)

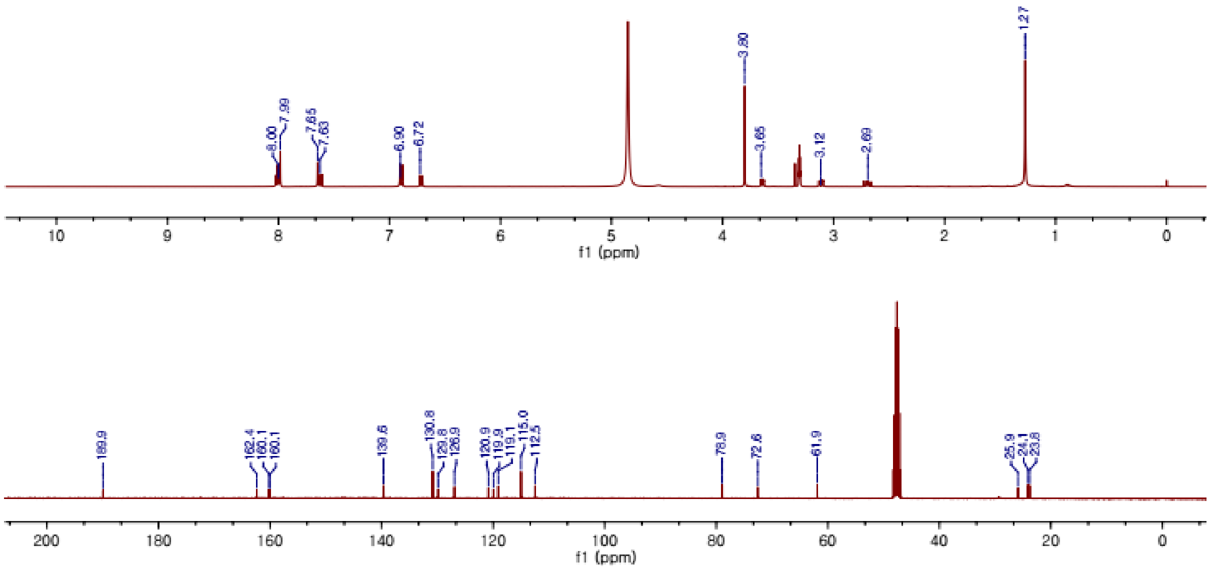

Figure S7. ^{1}H and ^{13}C NMR spectra of metabolite **6** (CD_3OD)

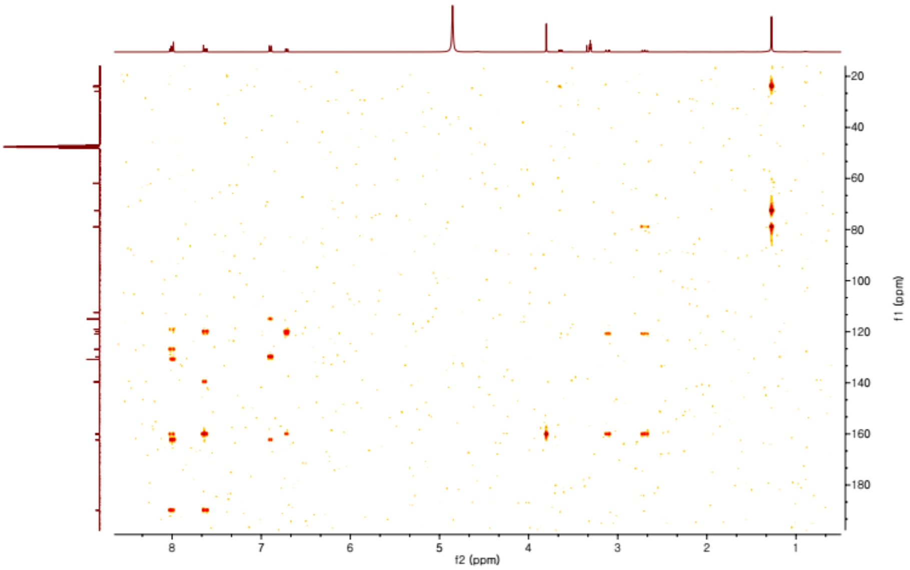

Figure S8. HMBC of metabolite **6** (CD_3OD)

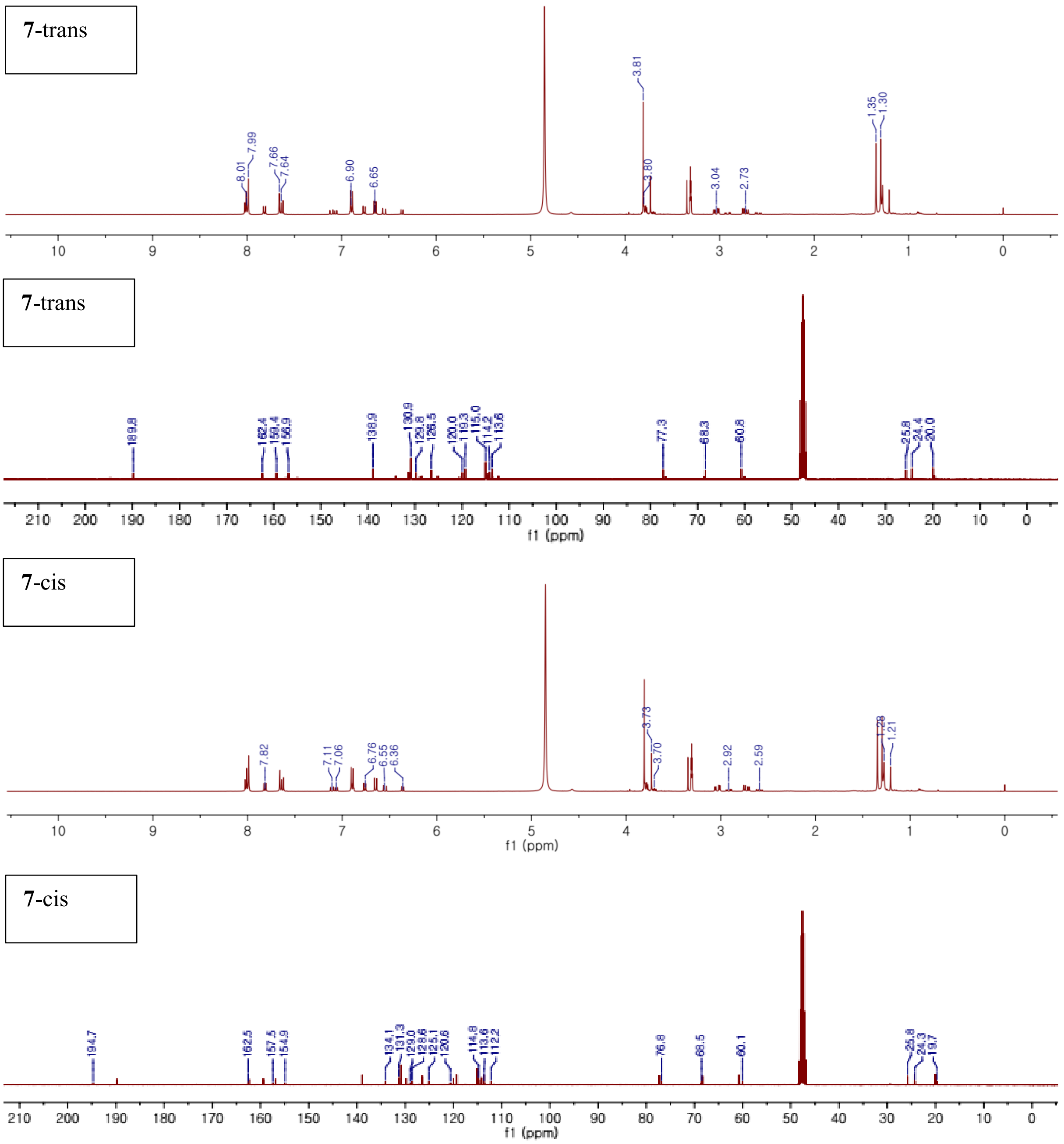

Figure S9. ^{1}H and ^{13}C NMR spectra of metabolite **7** (CD_3OD)

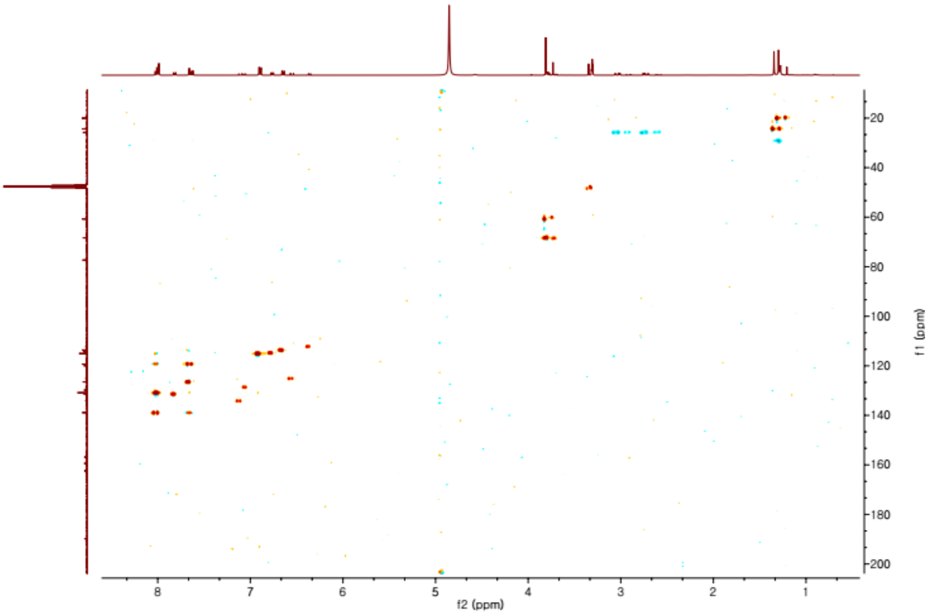

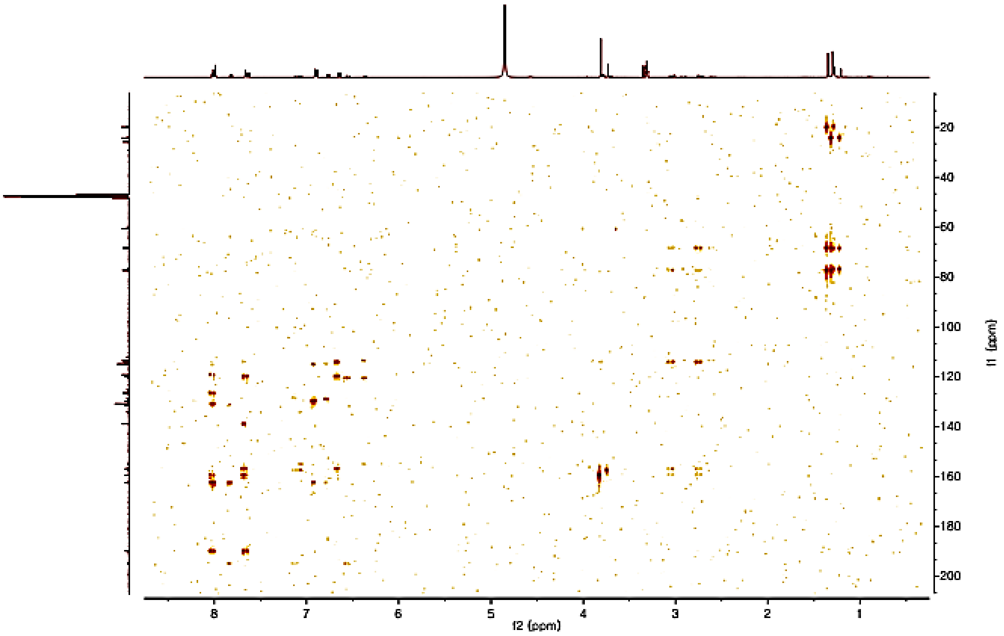

Figure S10. HSQC (up) and HMBC (down) of metabolite **7** (CD_3OD)

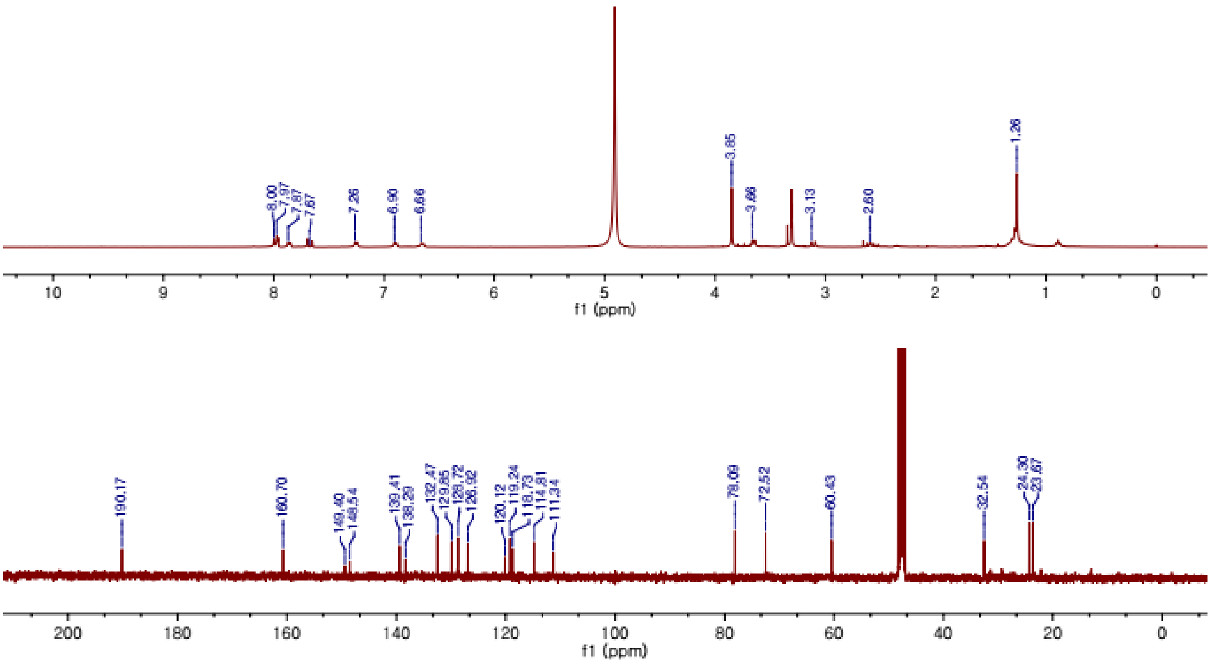

Figure S11. ^{1}H and ^{13}C NMR spectra of metabolite **8** (CD_3OD)

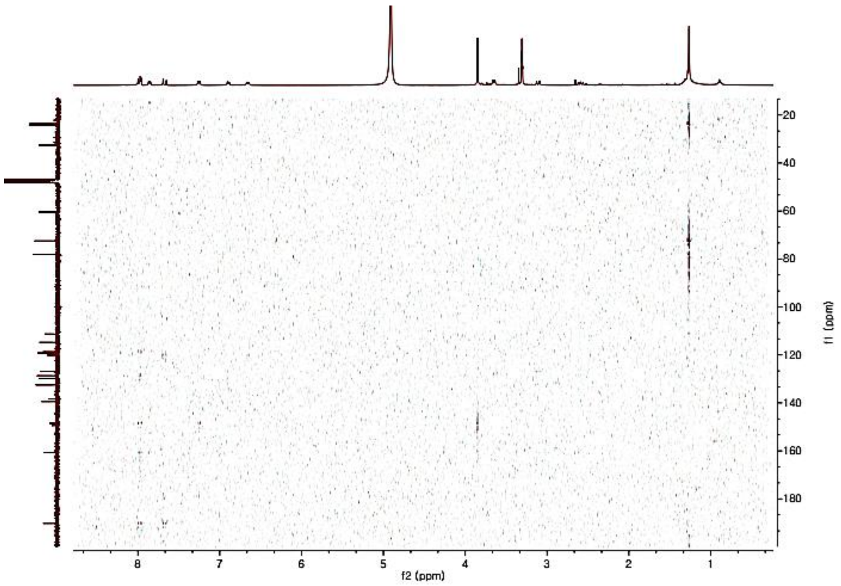

Figure S12. HMBC of metabolite **8** (CD_3OD)

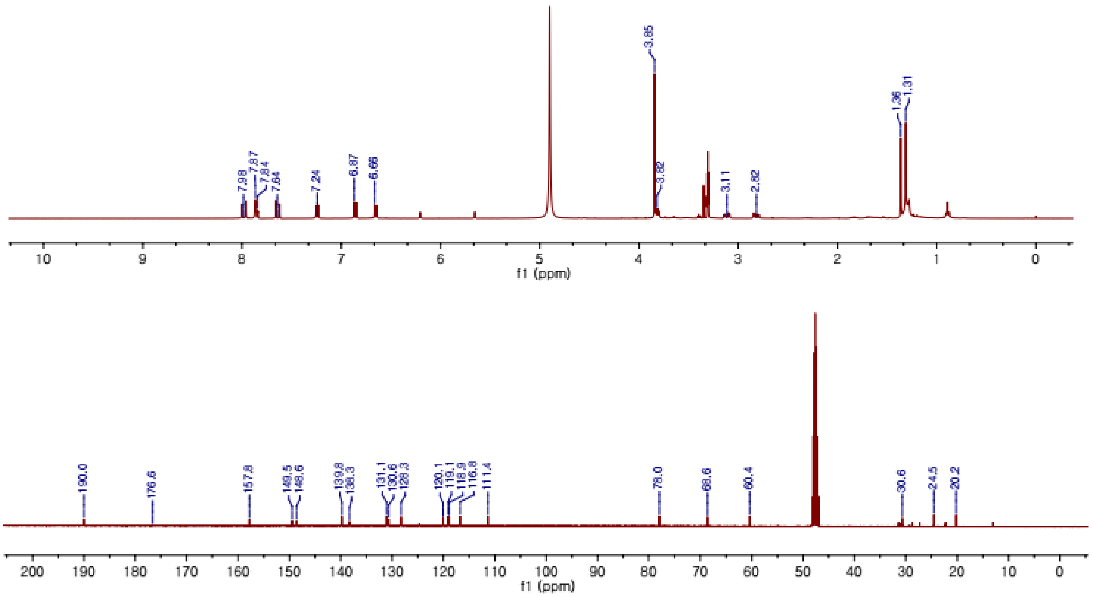

Figure S13. ^{1}H and ^{13}C NMR spectra of metabolite **9** (CD_3OD)

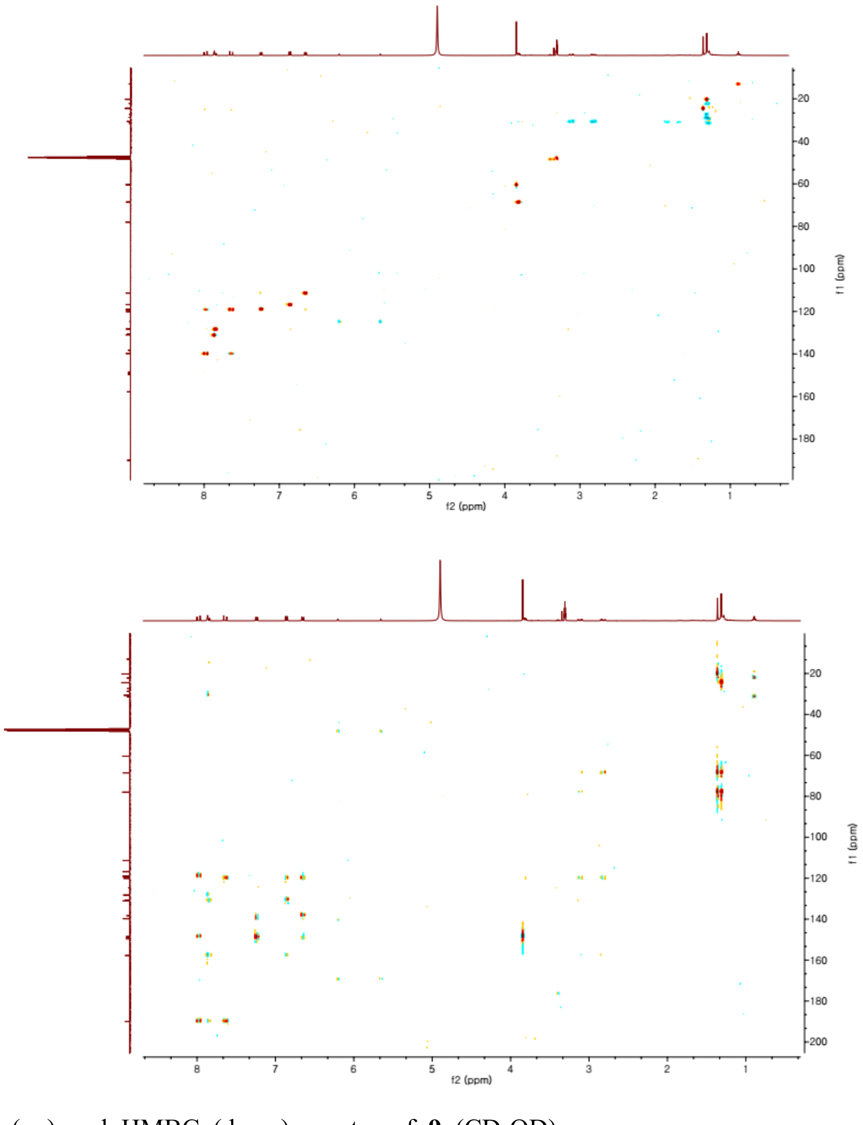

Figure S14. HSQC (up) and HMBC (down) spectra of **9** (CD_3OD)

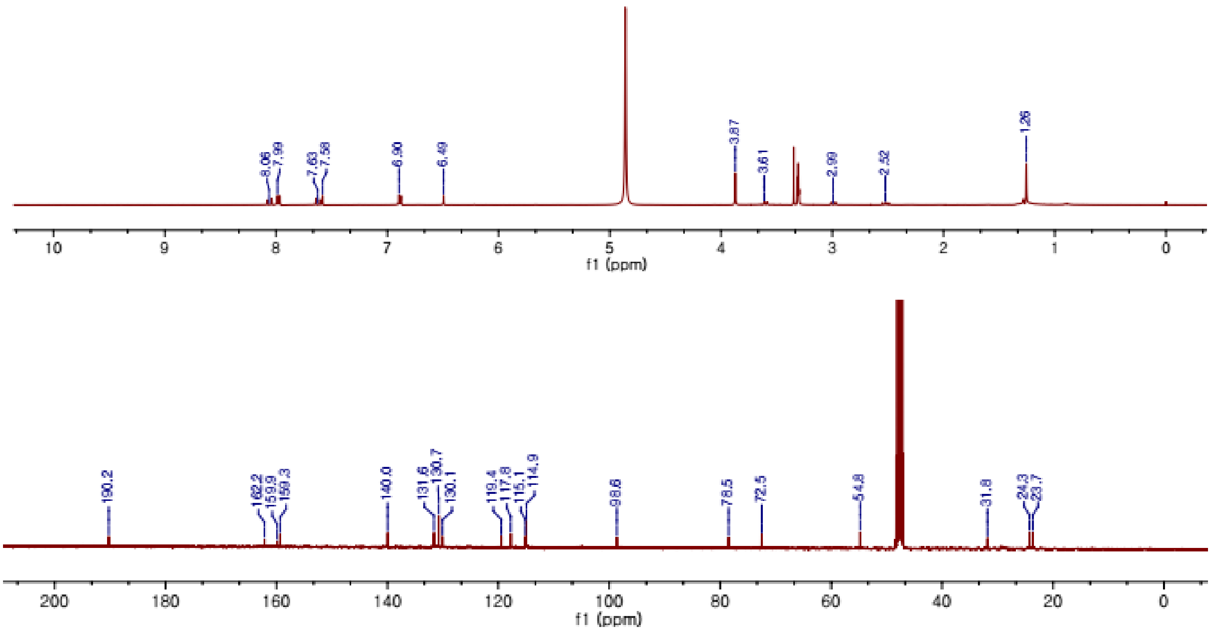

Figure S15. ^{1}H and ^{13}C NMR spectra of metabolite **10** (CD_3OD)

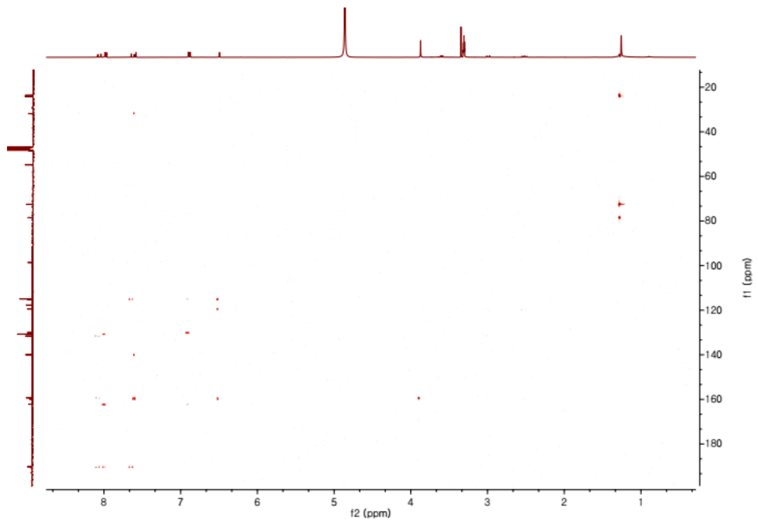

Figure S16. HMBC of metabolite **10** (CD_3OD)

11-trans

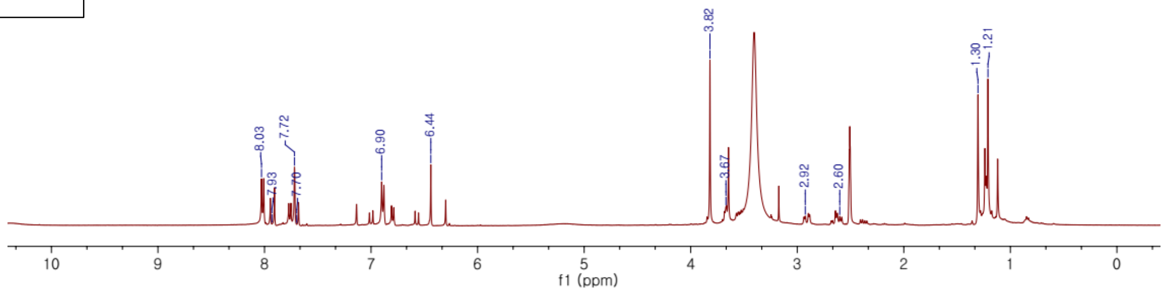

11-trans

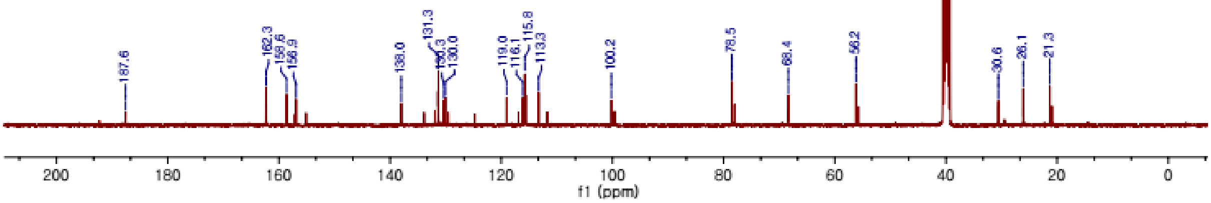

11-cis

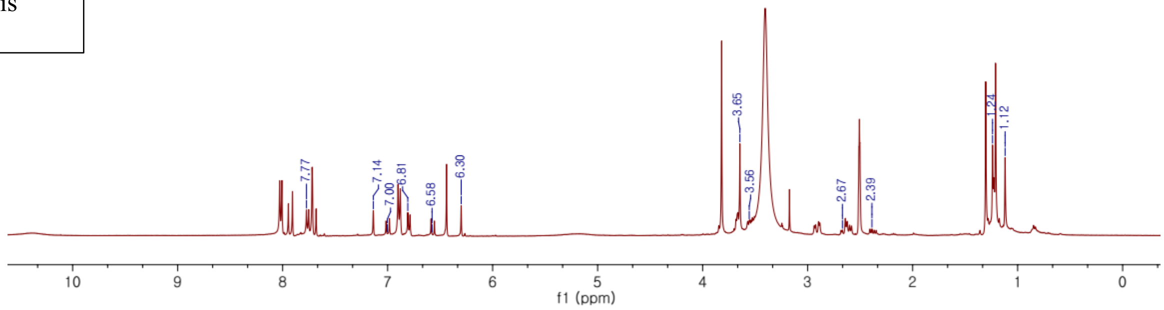

11-cis

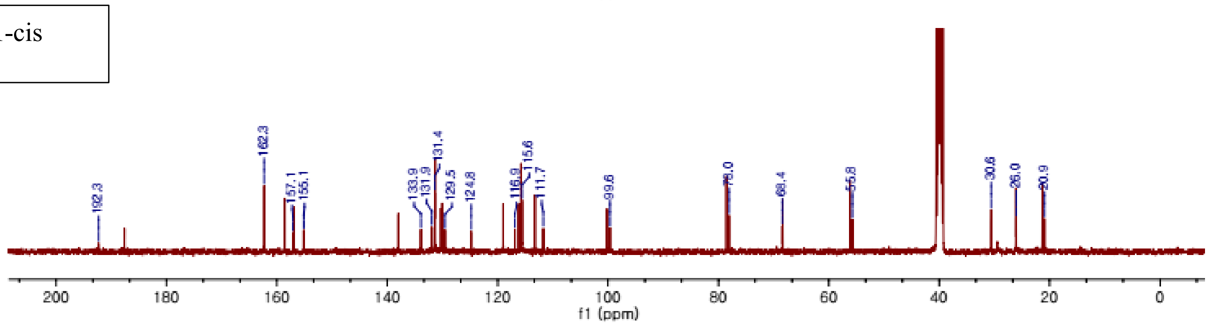

Figure S17. ^{1}H and ^{13}C NMR spectra of metabolite **11** (DMSO-d_6)

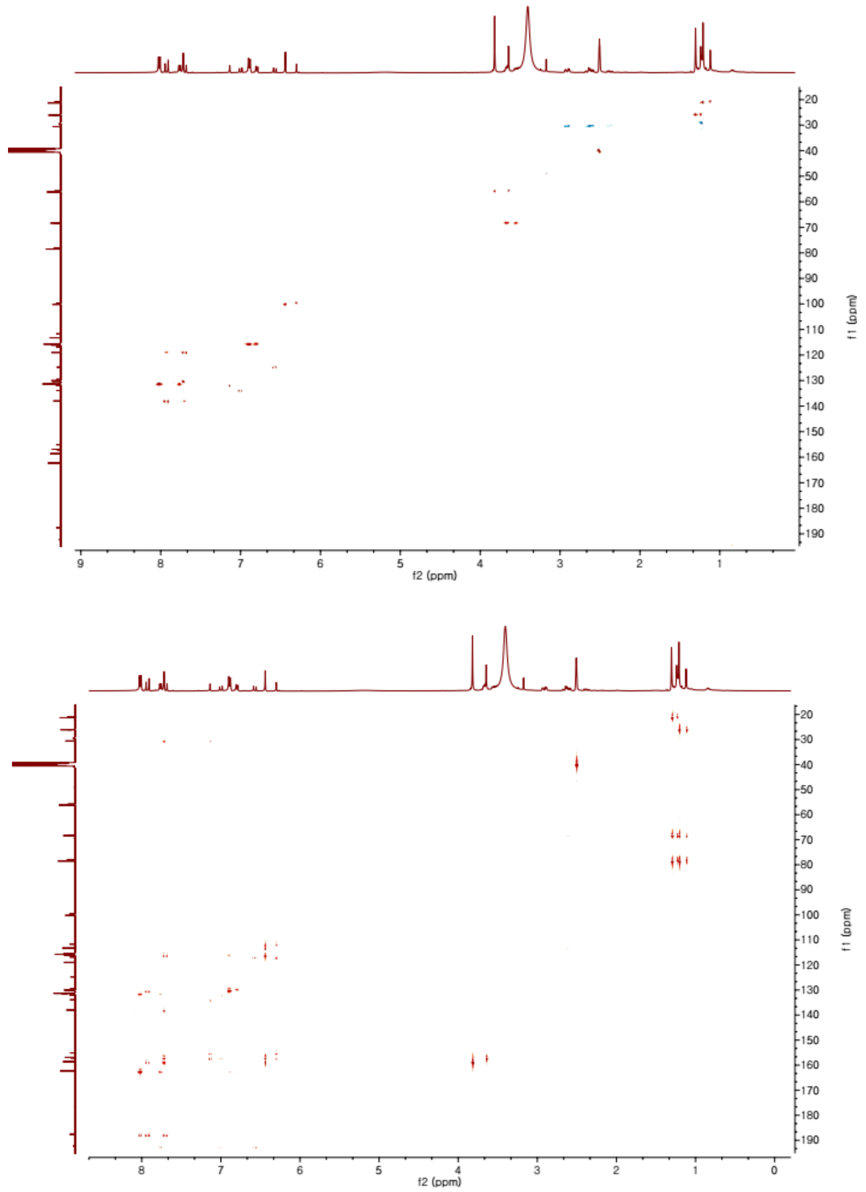

Figure S18. HSQC (up) and HMBC (down) of metabolite **11** (DMSO-d_6)

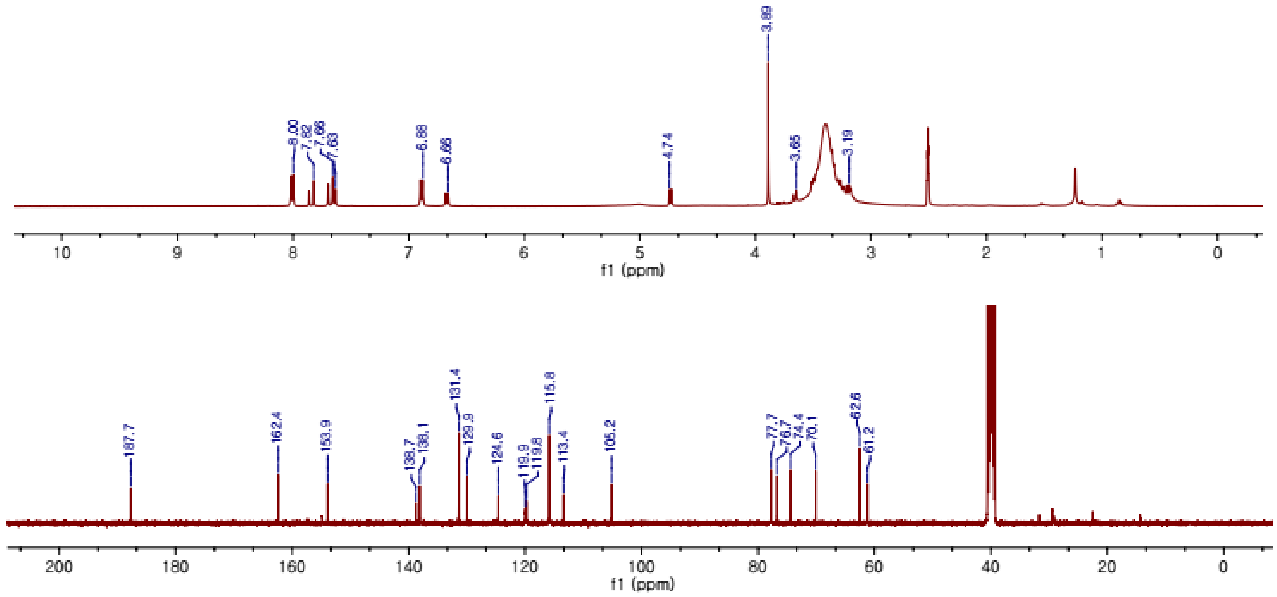

Figure S19. ^{1}H and ^{13}C NMR spectra of metabolite **12** (DMSO-d_6)

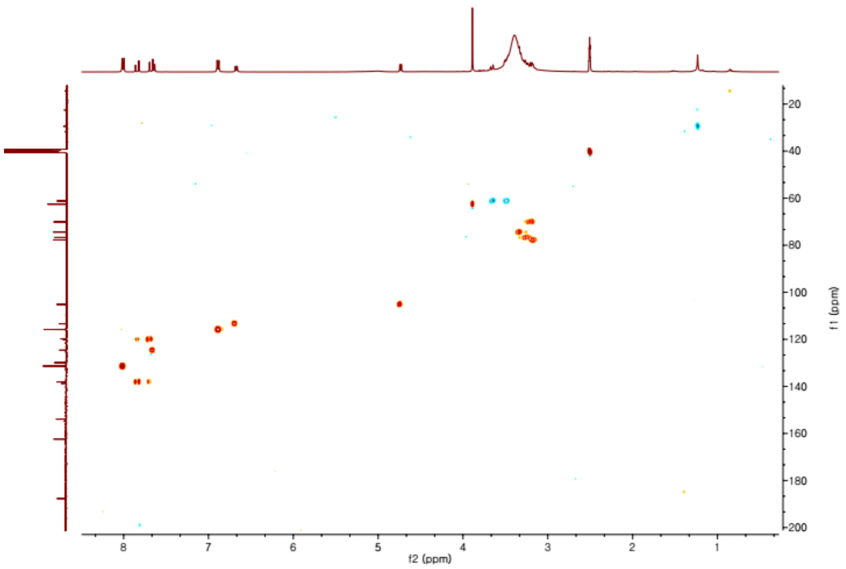

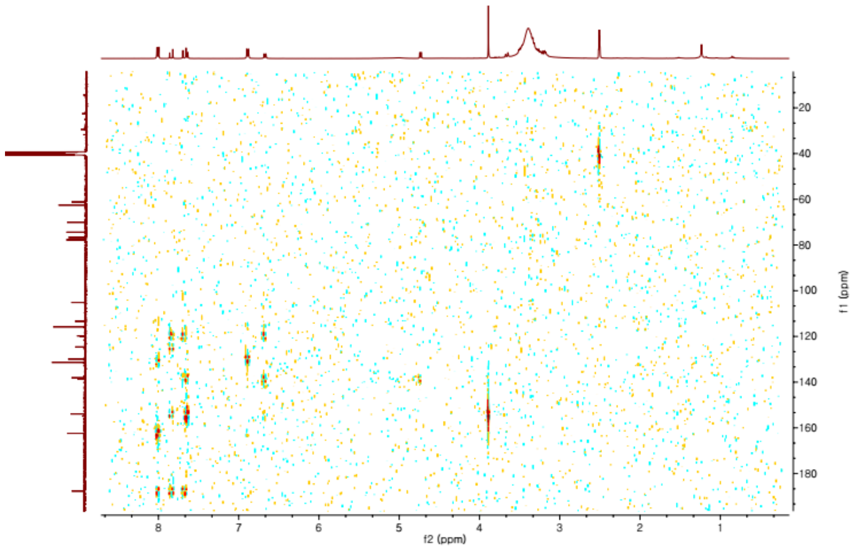

Figure S20. HSQC (up) and HMBC (down) of metabolite **12** (DMSO-d_6)

13-trans

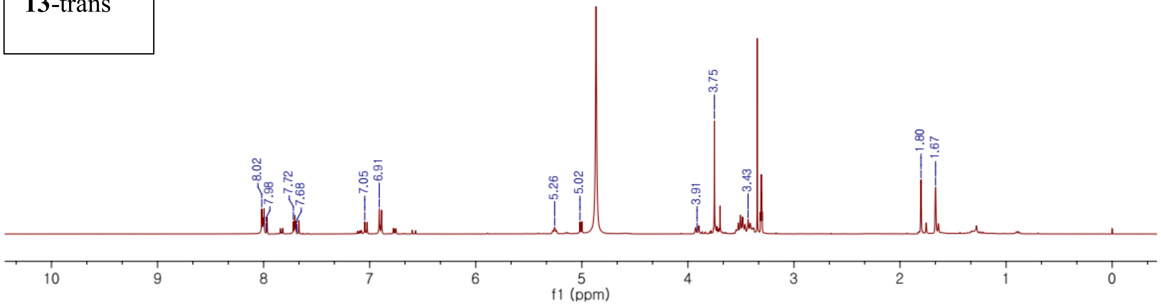

13-trans

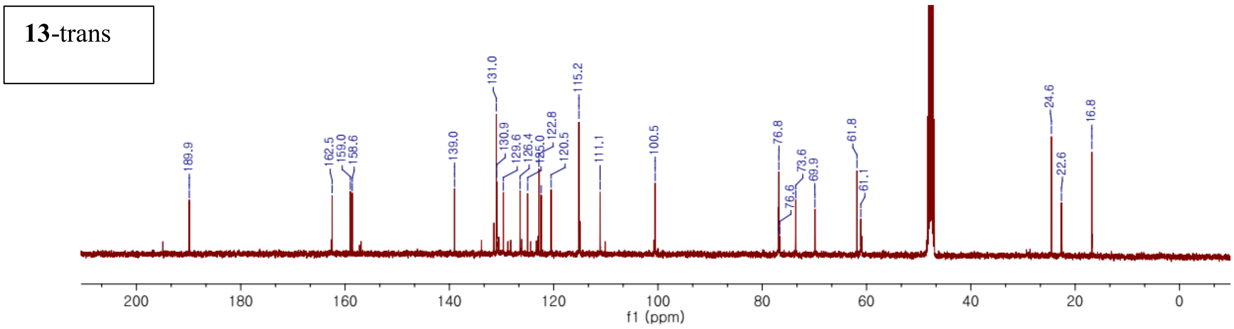

13-cis

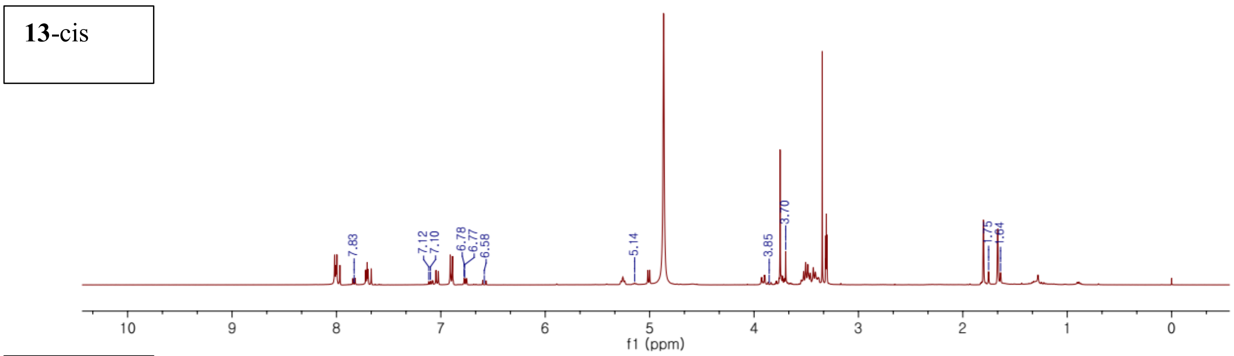

13-cis

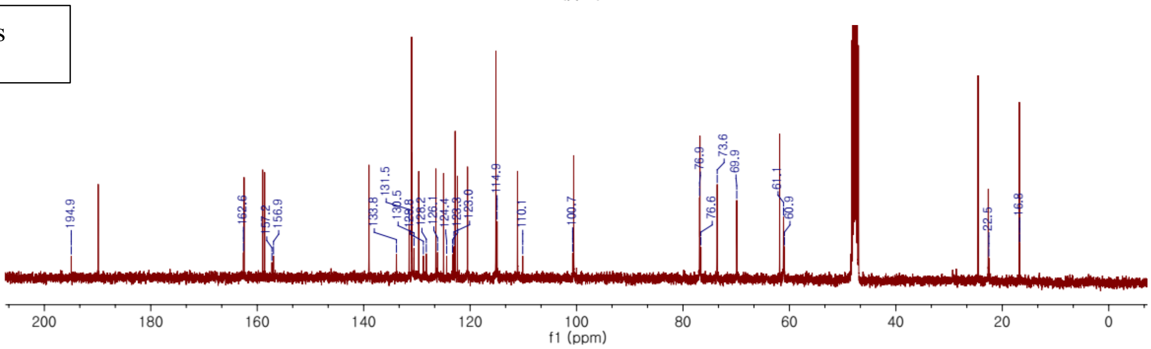

Figure S21. ^{1}H and ^{13}C NMR spectra of metabolite **13** (CD_3OD)

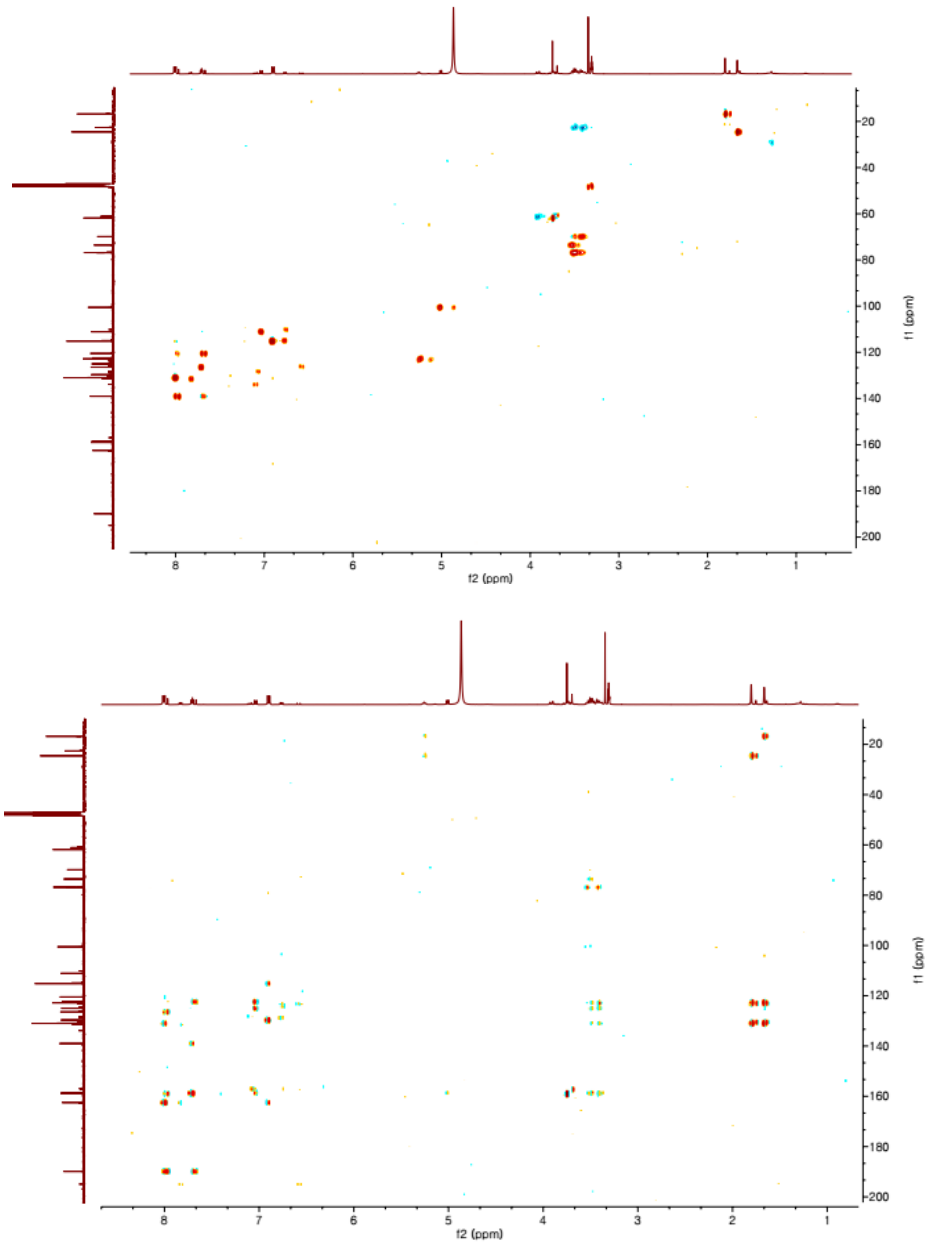

Figure S22. HSQC (up) and HMBC (down) of metabolite **13** (CD_3OD)

14-trans

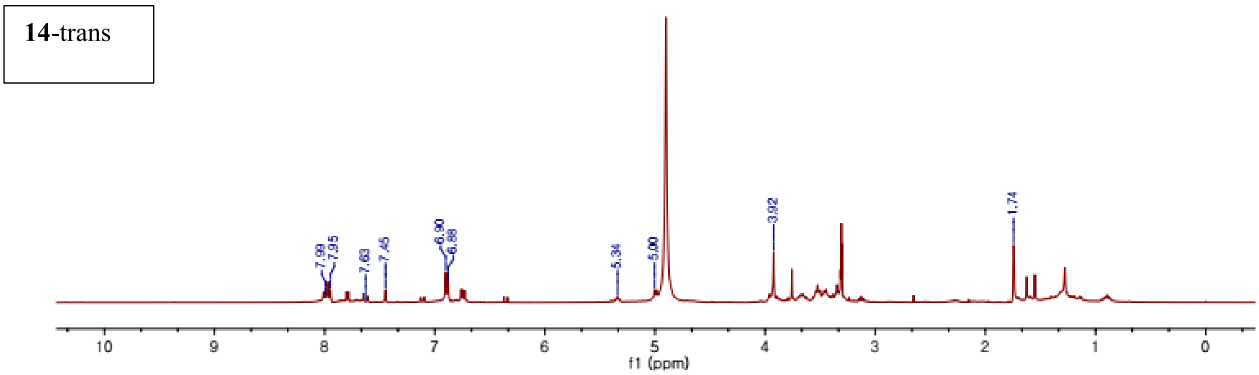

14-trans

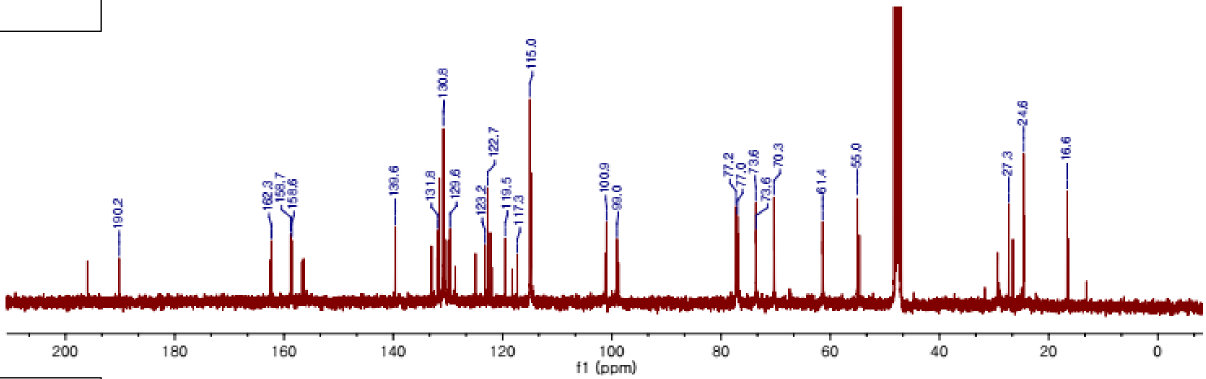

14-cis

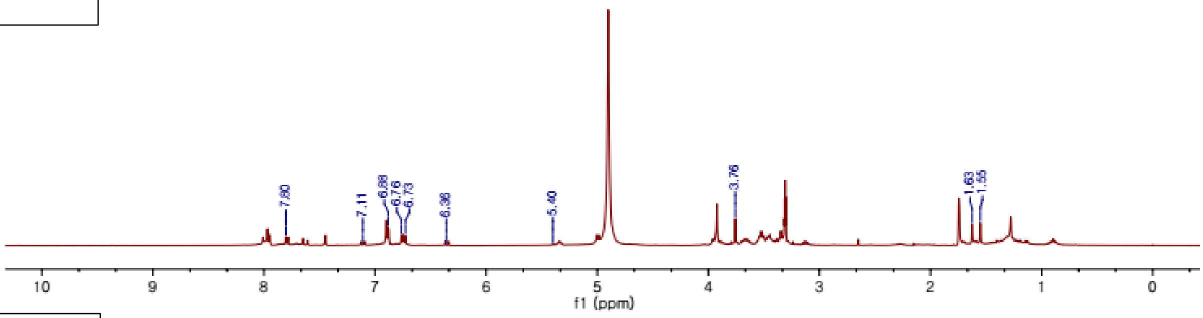

14-cis

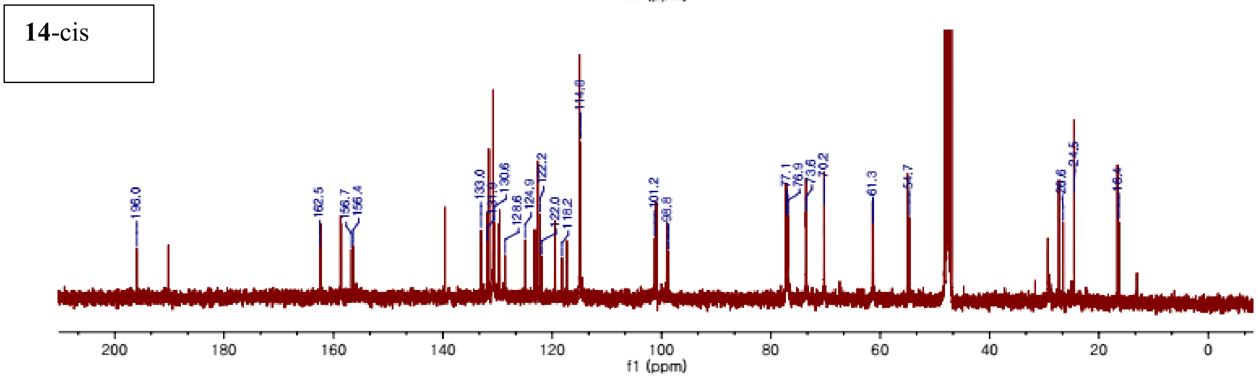

Figure S23. ^{1}H and ^{13}C NMR spectra of metabolite **14** (CD_3OD)

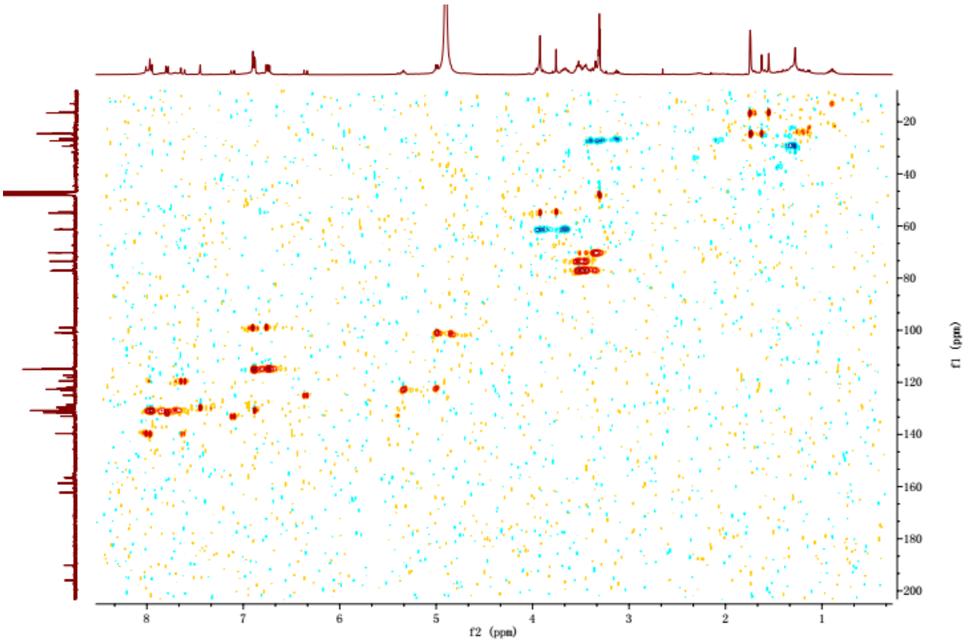

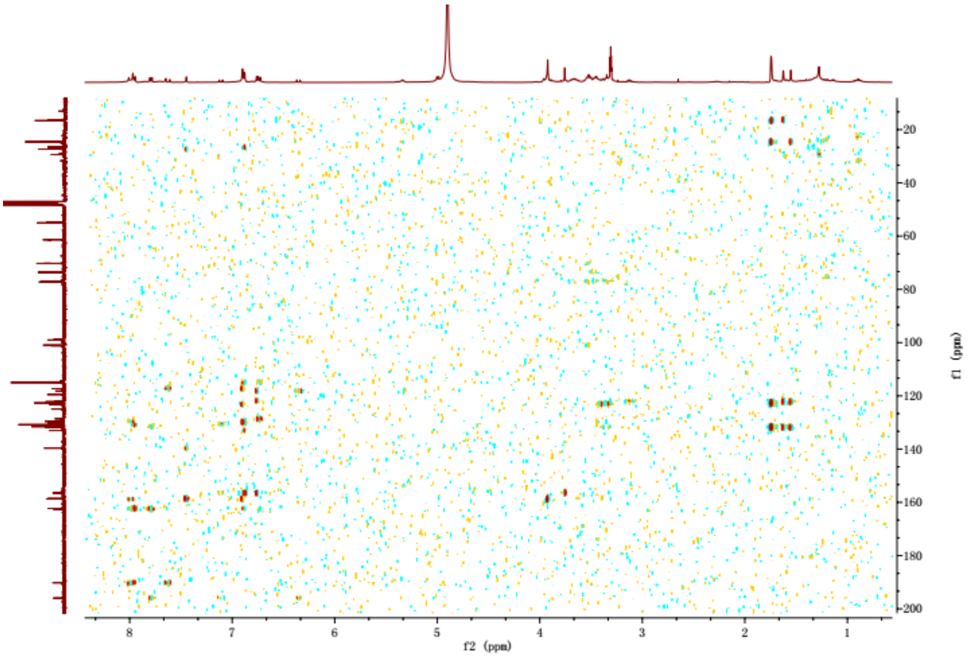

Figure S24. HSQC (up) and HMBC (down) of metabolite **14** (CD_3OD)

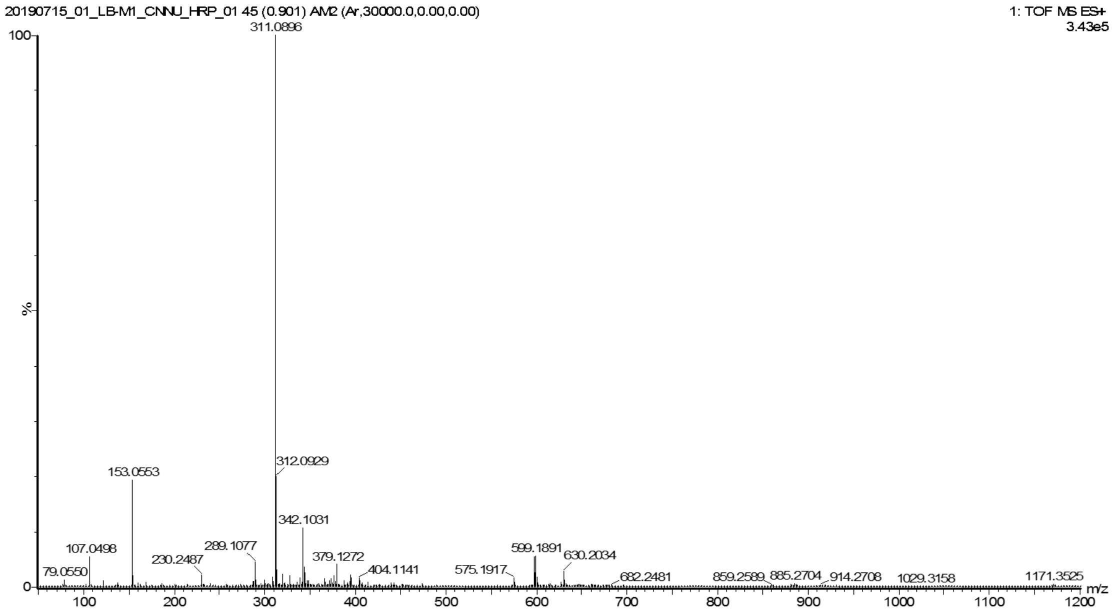

Elemental Composition Report

Single Mass Analysis
Tolerance = 5.0 PPM / DBE: min = -1.5, max = 100.0
Element prediction: Off
Number of isotope peaks used for i-FIT = 3

Monoisotopic Mass, Even Electron Ions
90 formula(e) evaluated with 1 results within limits (up to 50 closest results for each mass)
Elements Used:
C: 0-30 H: 0 -35 O: 0 -15 Na: 0 -1

Minimum:				-1.5				
Maximum:		5.0	5.0	100.0				
Mass	Calc. Mass	mDa	PPM	DBE	i-FIT	Norm	Conf(%)	Formula
311.0896	311.0895	0.1	0.3	8.5	961.1	n/a	n/a	C16 H16 O5 Na

Figure S25. High resolution ESIMS spectrum of metabolite **5**

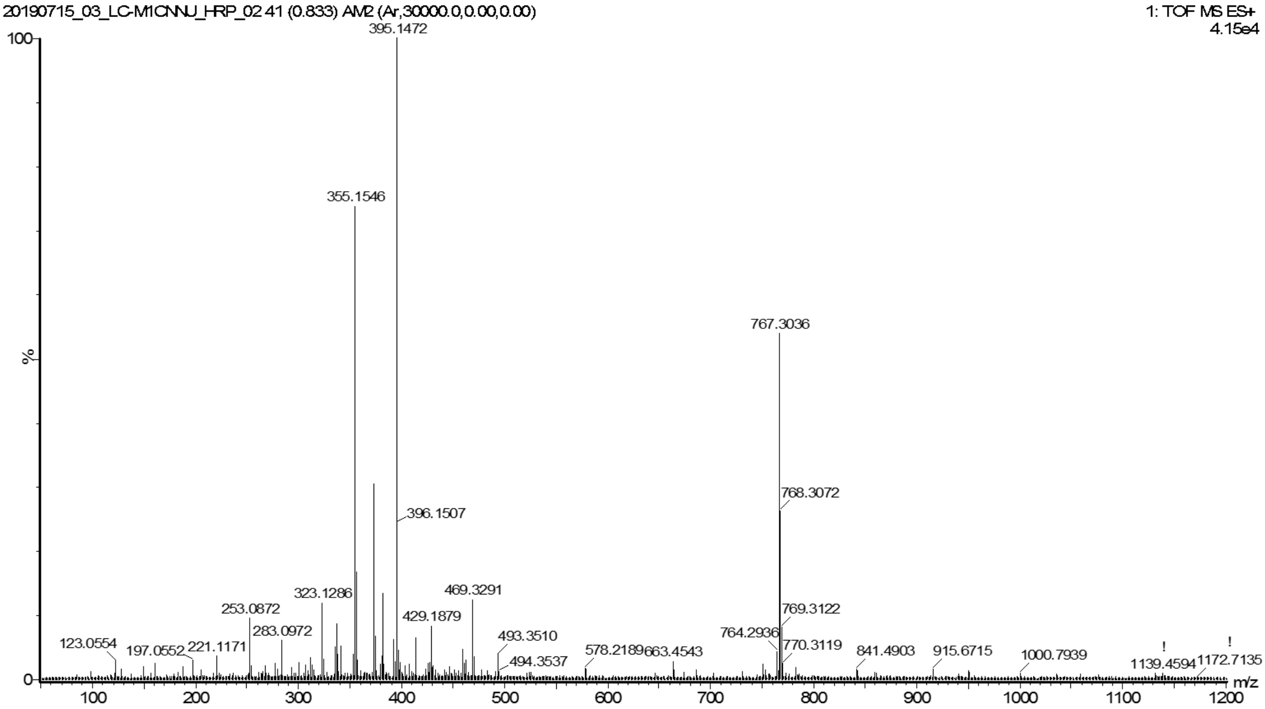

Elemental Composition Report

Single Mass Analysis
Tolerance = 5.0 PPM / DBE: min = -1.5, max = 100.0
Element prediction: Off
Number of isotope peaks used for i-FIT = 3

Monoisotopic Mass, Even Electron Ions
92 formula(e) evaluated with 1 results within limits (up to 50 closest results for each mass)
Elements Used:
C: 0-30 H: 0 -35 O: 0 -15 Na: 0 -1

Minimum:				-1.5				
Maximum:		5.0	5.0	100.0				
Mass	Calc. Mass	mDa	PPM	DBE	i-FIT	Norm	Conf(%)	Formula
373.1649	373.1651	-0.2	-0.5	9.5	456.6	n/a	n/a	C21 H25 O6
395.1472	395.1471	0.1	0.3	9.5	506.9	n/a	n/a	C21 H24 O6 Na

Figure S26. High resolution ESIMS spectrum of metabolite **6**

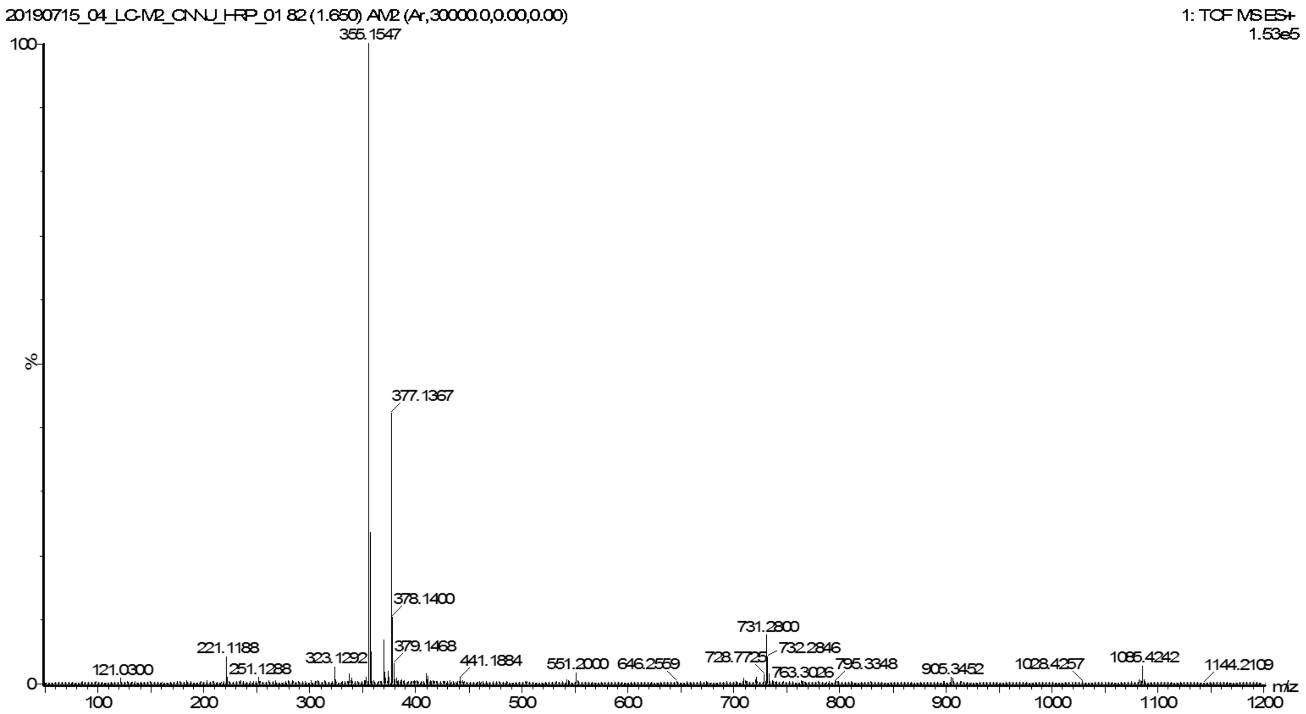

Elemental Composition Report

Single Mass Analysis
Tolerance = 3.0 PPM / DBE: min = -1.5, max = 100.0
Element prediction: Off
Number of isotope peaks used for i-FIT = 3

Monoisotopic Mass, Even Electron Ions
58 formula(e) evaluated with 1 results within limits (up to 50 closest results for each mass)
Elements Used:
C: 0-30 H: 0 -30 O: 0 -10 Na: 0 -1

Minimum:				-1.5				
Maximum:		100.0	3.0	100.0				
Mass	Calc. Mass	mDa	PPM	DBE	i-FIT	Norm	Conf(%)	Formula
355.1547	355.1545	0.2	0.6	10.5	749.6	n/a	n/a	C21 H23 O5
377.1367	377.1365	0.2	0.5	10.5	544.2	n/a	n/a	C21 H22 O5 Na

Figure S27. High resolution ESIMS spectrum of metabolite **7**

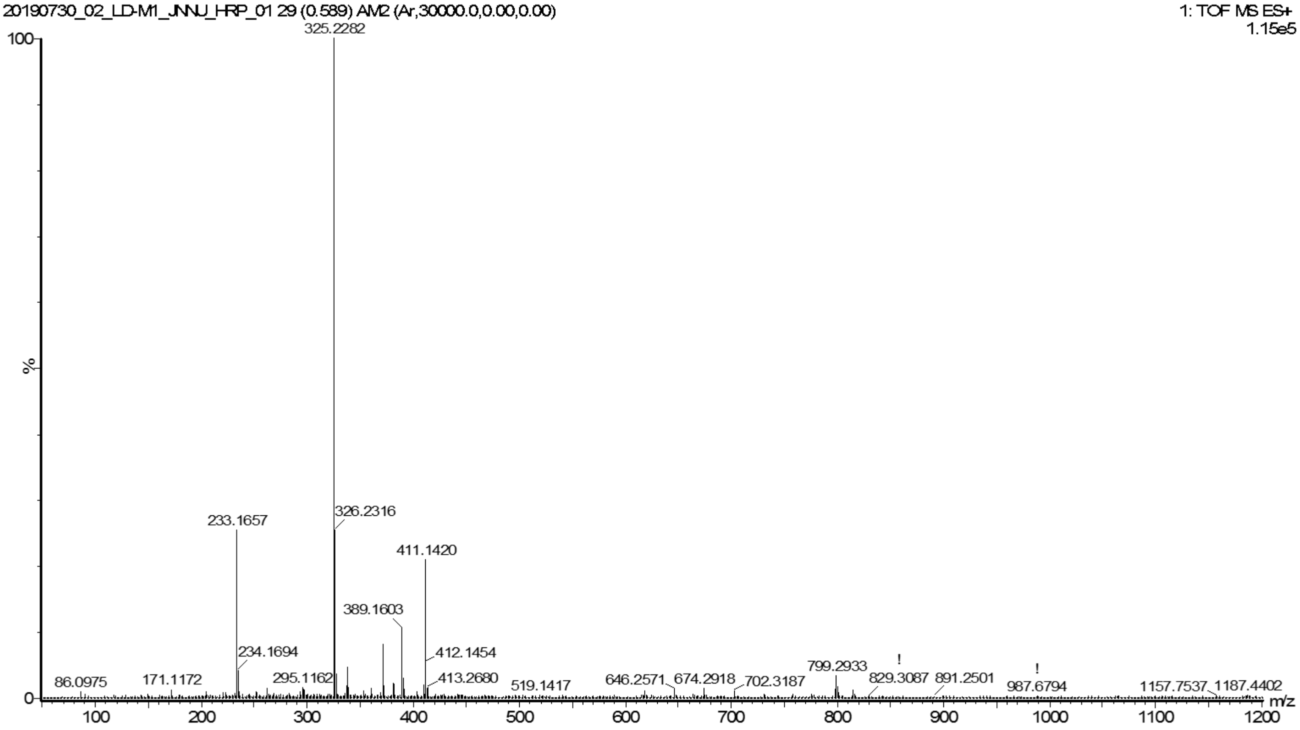

Elemental Composition Report

Single Mass Analysis
Tolerance = 5.0 PPM / DBE: min = -1.5, max = 100.0
Element prediction: Off
Number of isotope peaks used for i-FIT = 3

Monoisotopic Mass, Even Electron Ions
131 formula(e) evaluated with 1 results within limits (up to 50 closest results for each mass)
Elements Used:
C: 0-35 H: 0 -45 O: 0 -20 Na: 0 -1

Minimum:				-1.5				
Maximum:		100.0	5.0	100.0				
Mass	Calc. Mass	mDa	PPM	DBE	i-FIT	Norm	Conf(%)	Formula
389.1603	389.1600	0.3	0.8	9.5	394.8	n/a	n/a	C21 H25 O7

Figure S28. High resolution ESIMS spectrum of metabolite **8**

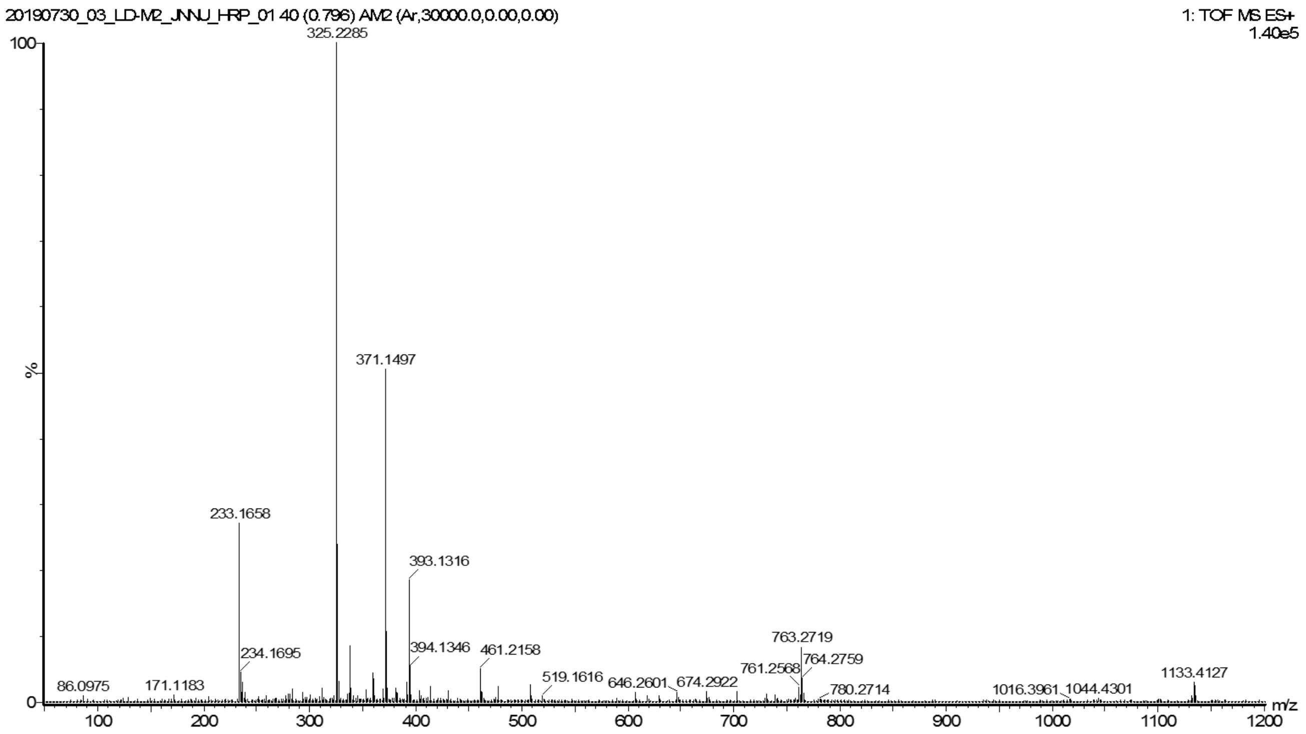

Elemental Composition Report

Single Mass Analysis
Tolerance = 5.0 PPM / DBE: min = -1.5, max = 100.0
Element prediction: Off
Number of isotope peaks used for i-FIT = 3

Monoisotopic Mass, Even Electron Ions
132 formula(e) evaluated with 1 results within limits (up to 50 closest results for each mass)
Elements Used:
C: 0-35 H: 0 -45 O: 0 -20 Na: 0 -1

Minimum:				-1.5				
Maximum:		100.0	5.0	100.0				
Mass	Calc. Mass	mDa	PPM	DBE	i-FIT	Norm	Conf(%)	Formula
371.1497	371.1495	0.2	0.5	10.5	689.5	n/a	n/a	C21 H23 O6

Figure S29. High resolution ESIMS spectrum of metabolite **9**

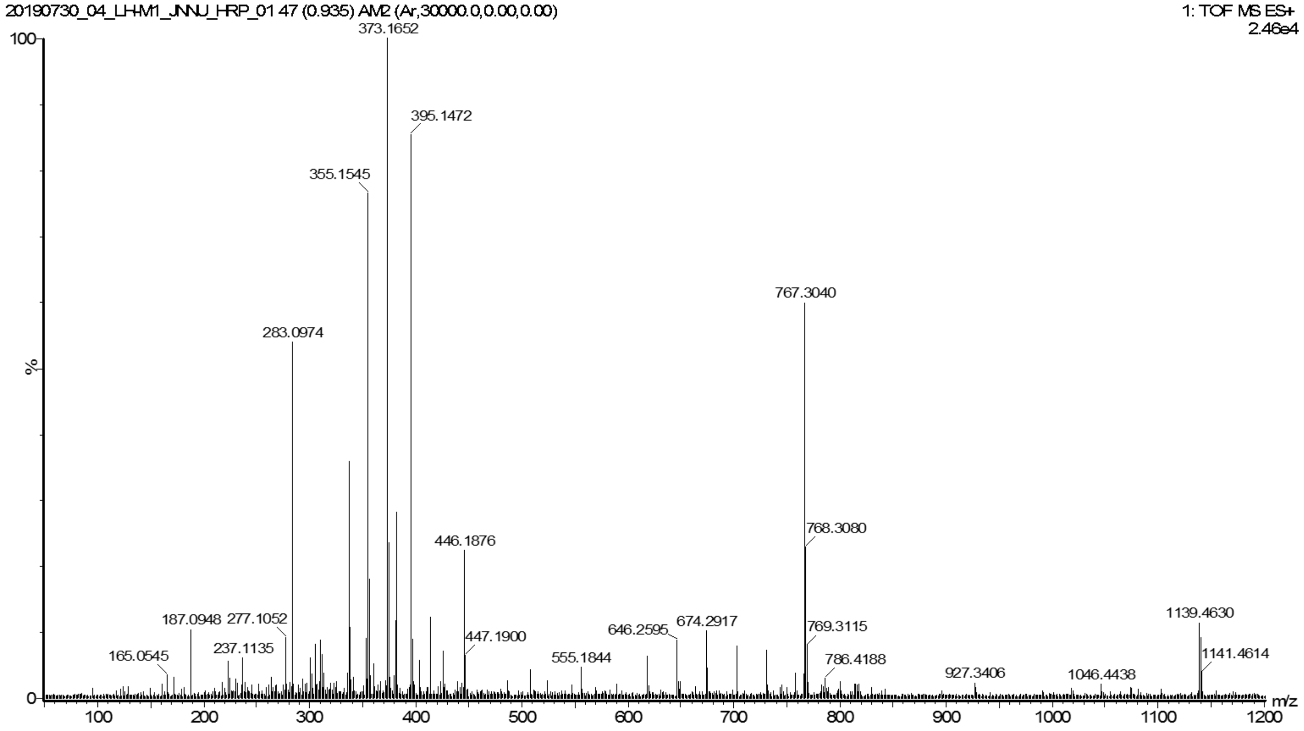

Elemental Composition Report

Single Mass Analysis
Tolerance = 5.0 PPM / DBE: min = -1.5, max = 100.0
Element prediction: Off
Number of isotope peaks used for i-FIT = 3

Monoisotopic Mass, Even Electron Ions
126 formula(e) evaluated with 1 results within limits (up to 50 closest results for each mass)
Elements Used:
C: 0-35 H: 0 -45 O: 0 -20 Na: 0 -1

Minimum:				-1.5				
Maximum:		100.0	5.0	100.0				
Mass	Calc. Mass	mDa	PPM	DBE	i-FIT	Norm	Conf(%)	Formula
373.1652	373.1651	0.1	0.3	9.5	529.6	n/a	n/a	C21 H25 O6

Figure S30. High resolution ESIMS spectrum of metabolite **10**

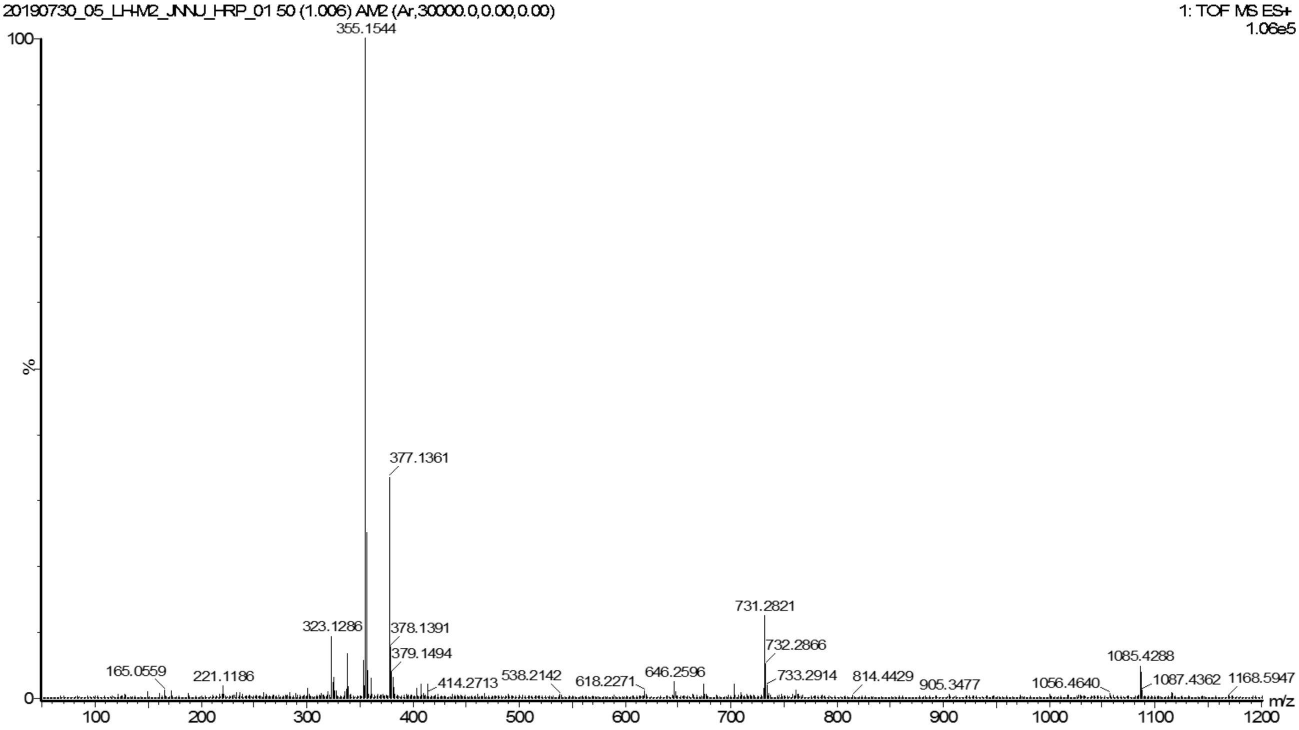

Elemental Composition Report

Single Mass Analysis
Tolerance = 5.0 PPM / DBE: min = -1.5, max = 100.0
Element prediction: Off
Number of isotope peaks used for i-FIT = 3

Monoisotopic Mass, Even Electron Ions
127 formula(e) evaluated with 1 results within limits (up to 50 closest results for each mass)
Elements Used:
C: 0-35 H: 0 -45 O: 0 -20 Na: 0 -1

Minimum:				-1.5				
Maximum:		100.0	5.0	100.0				
Mass	Calc. Mass	mDa	PPM	DBE	i-FIT	Norm	Conf(%)	Formula
355.1544	355.1545	-0.1	-0.3	10.5	822.3	n/a	n/a	C21 H23 O5

Figure S31. High resolution ESIMS spectrum of metabolite **11**

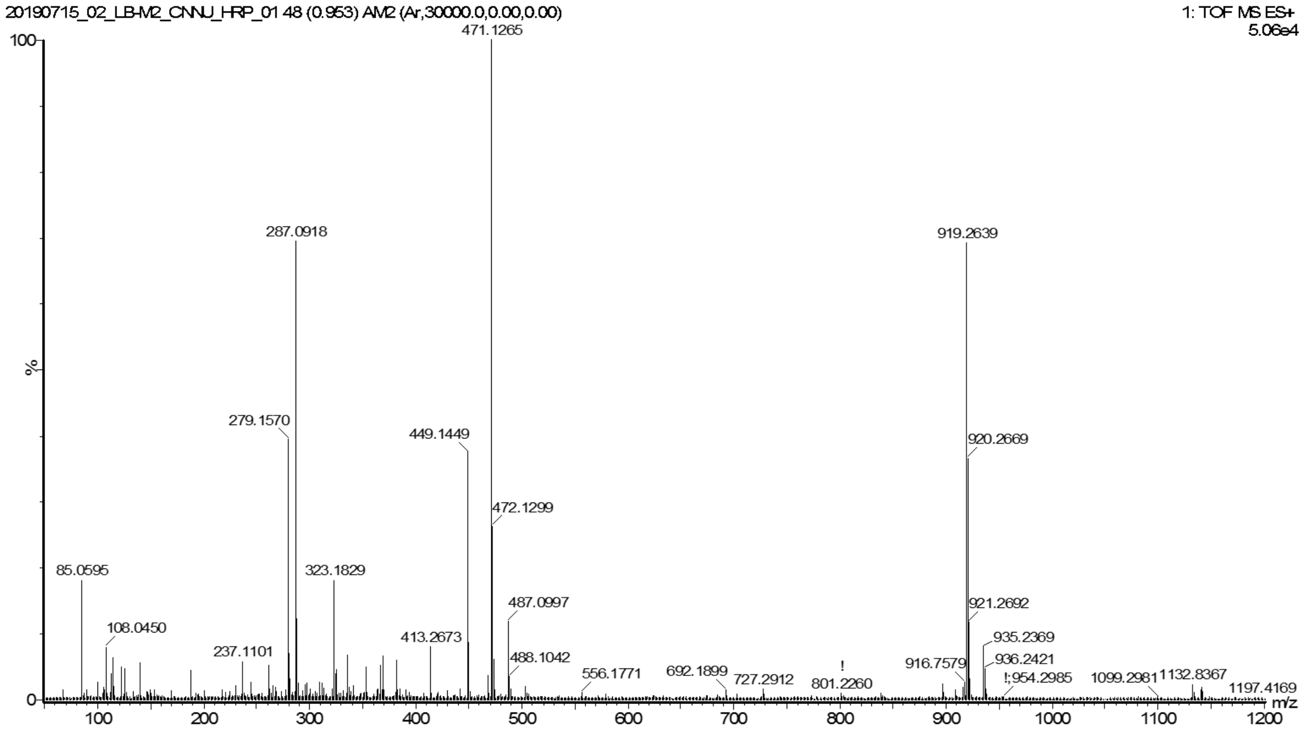

Elemental Composition Report

Single Mass Analysis
Tolerance = 5.0 PPM / DBE: min = -1.5, max = 100.0
Element prediction: Off
Number of isotope peaks used for i-FIT = 3

Monoisotopic Mass, Even Electron Ions
75 formula(e) evaluated with 1 results within limits (up to 50 closest results for each mass)
Elements Used:
C: 0-30 H: 0 -35 O: 0 -15 Na: 0 -1

Minimum:				-1.5				
Maximum:		5.0	5.0	100.0				
Mass	Calc. Mass	mDa	PPM	DBE	i-FIT	Norm	Conf(%)	Formula
449.1449	449.1448	0.1	0.2	10.5	333.7	n/a	n/a	C22 H25 O10
471.1265	471.1267	-0.2	-0.4	10.5	475.1	n/a	n/a	C22 H24 O10 Na

Figure S32. High resolution ESIMS spectrum of metabolite **12**

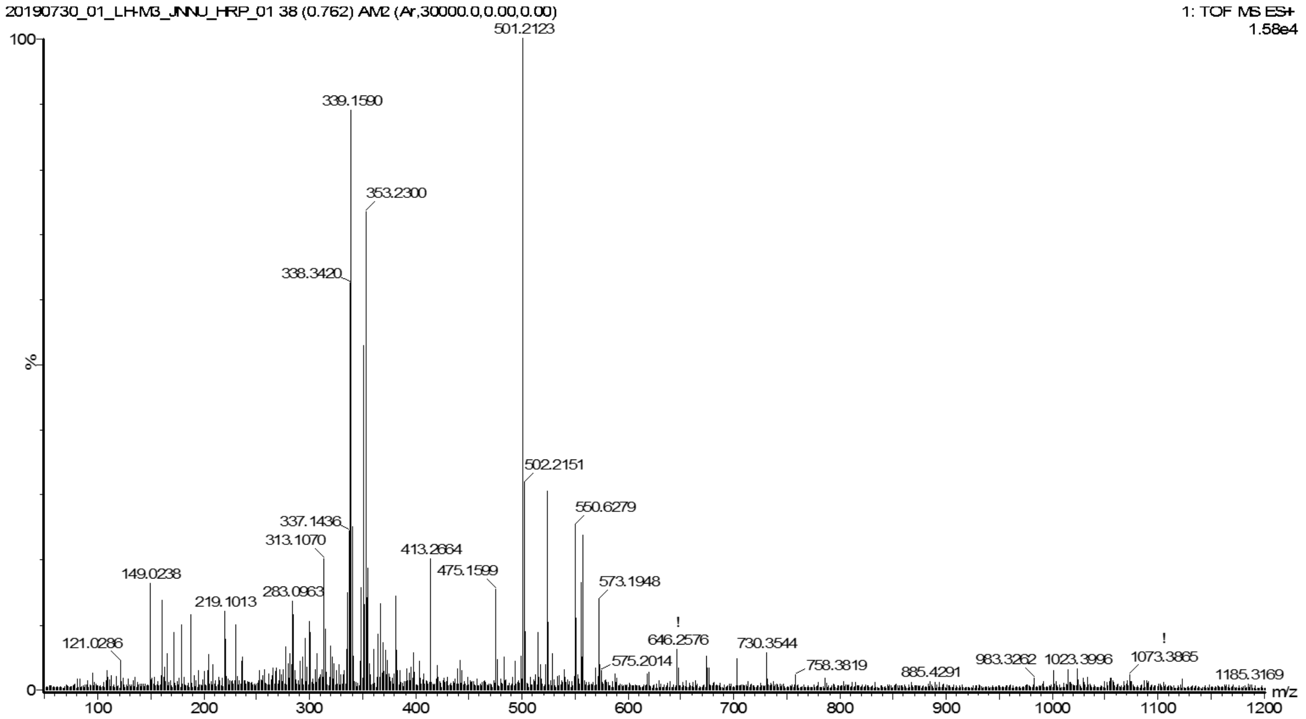

Elemental Composition Report

Single Mass Analysis
Tolerance = 5.0 PPM / DBE: min = -1.5, max = 100.0
Element prediction: Off
Number of isotope peaks used for i-FIT = 3

Monoisotopic Mass, Even Electron Ions
132 formula(e) evaluated with 2 results within limits (up to 50 closest results for each mass)
Elements Used:
C: 0-35 H: 0 -45 O: 0 -20 Na: 0 -1

Minimum:				-1.5				
Maximum:		100.0	5.0	100.0				
Mass	Calc. Mass	mDa	PPM	DBE	i-FIT	Norm	Conf(%)	Formula
501.2123	501.2125	-0.2	-0.4	11.5	356.5	0.091	91.33	C27 H33 O9
	501.2101	2.2	4.4	8.5	358.9	2.445	8.67	C25 H34 O9 Na

Figure S33. High resolution ESIMS spectrum of metabolite **13**

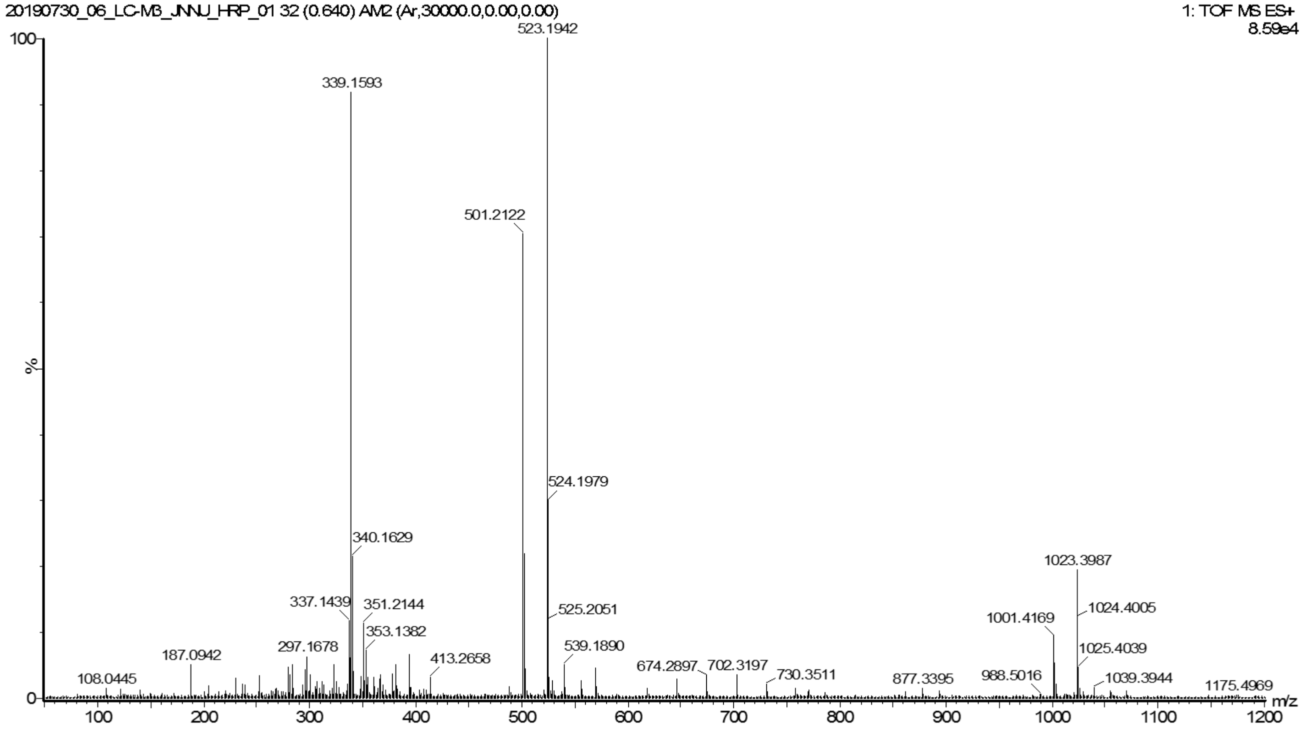

Elemental Composition Report

Single Mass Analysis
Tolerance = 5.0 PPM / DBE: min = -1.5, max = 100.0
Element prediction: Off
Number of isotope peaks used for i-FIT = 3

Monoisotopic Mass, Even Electron Ions
132 formula(e) evaluated with 2 results within limits (up to 50 closest results for each mass)
Elements Used:
C: 0-35 H: 0 -45 O: 0 -20 Na: 0 -1

Minimum:				-1.5				
Maximum:		100.0	5.0	100.0				
Mass	Calc. Mass	mDa	PPM	DBE	i-FIT	Norm	Conf(%)	Formula
501.2122	501.2125	-0.3	-0.6	11.5	542.9	0.003	99.70	C27 H33 O9
	501.2101	2.1	4.2	8.5	548.7	5.809	0.30	C25 H34 O9 Na

Figure S34. High resolution ESIMS spectrum of metabolite **14**

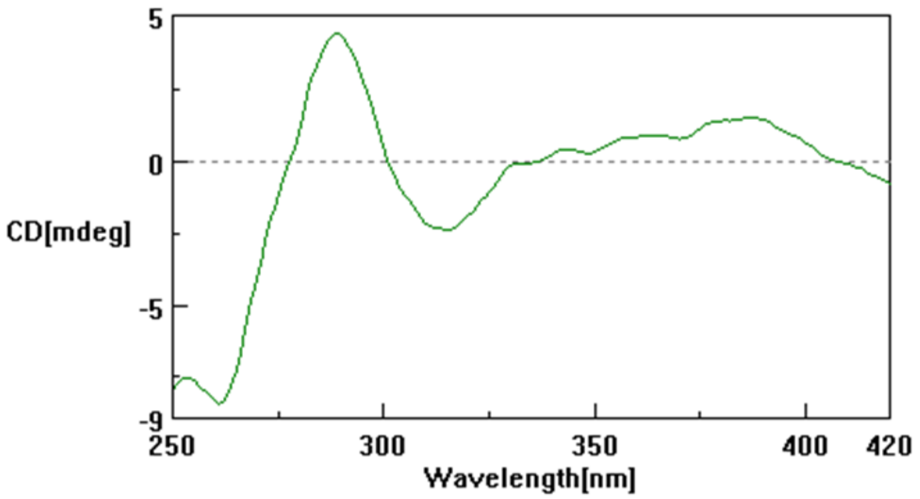

Figure S35. ICD spectrum of the Mo-complex of **8**

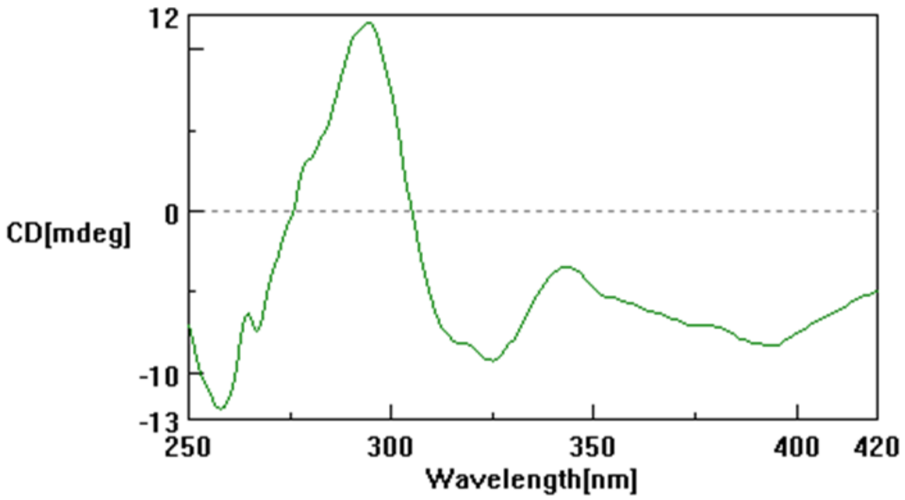

Figure S36. ICD spectrum of the Mo-complex of **10**
